# Curcumin, a Natural Antimicrobial Agent with Strain-Specific Activity

**DOI:** 10.3390/ph13070153

**Published:** 2020-07-16

**Authors:** Artur Adamczak, Marcin Ożarowski, Tomasz M. Karpiński

**Affiliations:** 1 Department of Botany, Breeding and Agricultural Technology of Medicinal Plants, Institute of Natural Fibres and Medicinal Plants, Kolejowa 2, 62-064 Plewiska, Poland; artur.adamczak@iwnirz.pl; 2 Department of Biotechnology, Institute of Natural Fibres and Medicinal Plants, Wojska Polskiego 71b, 60-630 Poznań, Poland; marcin.ozarowski@iwnirz.pl; 3 Chair and Department of Medical Microbiology, Poznań University of Medical Sciences, Wieniawskiego 3, 61-712 Poznań, Poland

**Keywords:** *Curcuma longa*, curcumin, antibacterial activity, antifungal activity, minimum inhibitory concentration (MIC), multidrug-resistant (MDR) strains

## Abstract

Curcumin, a principal bioactive substance of turmeric (*Curcuma longa* L.), is reported as a strong antioxidant, anti-inflammatory, antibacterial, antifungal, and antiviral agent. However, its antimicrobial properties require further detailed investigations into clinical and multidrug-resistant (MDR) isolates. In this work, we tested curcumin’s efficacy against over 100 strains of pathogens belonging to 19 species. This activity was determined by the broth microdilution method and by calculating the minimum inhibitory concentration (MIC). Our findings confirmed a much greater sensitivity of Gram-positive than Gram-negative bacteria. This study exhibited a significantly larger variation in the curcumin activity than previous works and suggested that numerous clinical strains of widespread pathogens have a poor sensitivity to curcumin. Similarly, the MICs of the MDR types of *Staphylococcus aureus*, *S. haemolyticus*, *Escherichia coli*, and *Proteus mirabilis* were high (≥2000 µg/mL). However, curcumin was effective against some species and strains: *Streptococcus pyogenes* (median MIC = 31.25 µg/mL), methicillin-sensitive *S. aureus* (250 µg/mL), *Acinetobacter lwoffii* (250 µg/mL), and individual strains of *Enterococcus faecalis* and *Pseudomonas aeruginosa* (62.5 µg/mL). The sensitivity of species was not associated with its affiliation to the genus, and it could differ a lot (e.g., *S. pyogenes*, *S. agalactiae* and *A. lwoffii*, *A. baumannii*). Hence, curcumin can be considered as a promising antibacterial agent, but with a very selective activity.

## 1. Introduction

**Figure 1:**
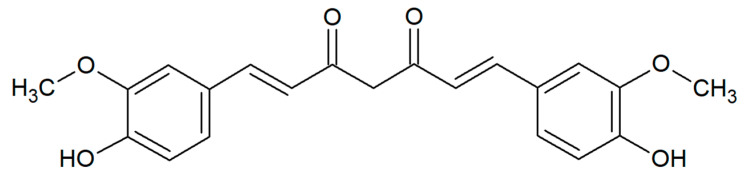
Chemical structure of curcumin.

Curcumin (curcumin I, diferuloylmethane) is a dimeric derivative of ferulic acid, composed of two *o*-methoxyphenol rings connected by a heptadienedione chain (Figure 1). It has a chemical formula of C_21_H_20_O_6_ and a molecular weight of 368.38 g/mol. This lipophilic polyphenol is a natural pigment with a characteristic yellow–orange color, predominantly found in the rhizomes of turmeric (*Curcuma longa* L.) from the ginger family, Zingiberaceae, native to tropical South Asia. Together with essential oils and other curcuminoids, curcumin is a principal bioactive compound of turmeric powder—an oriental spice commonly obtained from this plant [1,2,3]. It is very popular in South Asian and Middle Eastern cuisines, especially for preparing curry dishes. The use of *C. longa* as a culinary spice and in religious ceremonies dates back nearly 4000 years to the Vedic culture in India. This plant has been also well known in Ayurvedic and Unani systems, Traditional Chinese Medicine (TCM), and in the folk medicine of Pakistan, Bangladesh, and Afghanistan. Turmeric has been traditionally used as an antiseptic, antibacterial, anti-inflammatory, choleretic, and carminative agent in the treatment of wounds and burns, gastrointestinal and liver disorders, respiratory system diseases (e.g., asthma, cough, runny nose, sinusitis), anorexia, and rheumatism [4,5]. Nowadays, turmeric and curcumin (the code of E100) are widely utilized as food additives with coloring, flavoring, and preservative properties (e.g., in mustard, margarine, butter, cheese, pasta, and beverages) [1,4]. Traditionally, curcumin is very often used to relieve many symptoms of various gastrointestinal diseases, such as diarrhea, indigestion, efflux, and even gastric and duodenal ulcers [6]. It is also able to diminish adverse effects after medication, i.e., through mucosal protection from the gastric damage induced by non-steroidal anti-inflammatory drugs [7,8].

Numerous in vitro and in vivo studies have confirmed the health-promoting effects of curcumin associated primarily with its strong antioxidant and anti-inflammatory activities [9,10,11]. This natural molecule also exhibits antibacterial, antifungal, antiviral, antiprotozoal, and antiparasitic properties [12,13,14]. Clinical trials have demonstrated the therapeutic benefits of curcumin supplementation in patients with inflammatory diseases (arthritis, inflammatory bowel disease, peptic ulcer, and *H. pylori* infection), metabolic syndrome, neurodegenerative diseases, and cancer, including colorectal, pancreatic, and breast cancers [11,15,16,17,18]. Due to the wide range of biological activities of curcumin and its pleiotropic therapeutic potential, this substance has been of great interest to researchers. According to the PubMed/MEDLINE database, 526 items with the keyword *curcumin* in the title/abstract were published in 1949–2000. However, a rapid increase in the number of these studies has been observed in the last two decades. There were 13,144 publications in 2001–2019, including 1863 works only in 2019.

The antibacterial activity of curcumin was first shown in *Nature* in 1949 [19]. In 1974, researchers from our Institute [20] published in the *Planta Medica* journal rich data on the effects of curcumin, an ethanol extract and essential oil of the rhizome of *C. longa* against 65 reference and clinical strains representing 56 bacterial and fungal taxa. They documented a high in vitro efficacy of curcumin against Gram-positive cocci (*Staphylococcus aureus*, *S. epidermidis*, *Streptococcus pyogenes*, *Micrococcus tetragenus*, *M. luteus*), spore-forming bacilli (*Bacillus* and *Clostridium* species), some Gram-negative bacteria (*Acinetobacter lwoffii*, *Alcaligenes faecalis*), and fungi (e.g., *Candida stellatoidea*, *Cryptococcus neoformans*, *Microsporum gypseum*, *Saccharomyces cerevisiae*, *Scopulariopsis brevicaulis*).

Modern studies have confirmed the strong antimicrobial potential of curcumin despite its poor solubility in water, low bioavailability and pharmacokinetic profile [3]. Curcumin has been reported for its antibiofilm activity through the inhibition of bacterial quorum sensing (QS) systems and removal of already formed biofilms [21,22]. This plant molecule was found to have a photodynamic action by the cytotoxic reactive oxygen species (ROS) production against both planktonic and biofilm forms of bacteria [23]. The literature data have also shown its beneficial effects against Gram-negative uropathogens (*Escherichia coli*, *Pseudomonas aeruginosa*, *Proteus mirabilis*, and *Serratia marcescens* [24]), and a preventive role in the formation of struvite stones associated with the urinary tract infections [25]. Moreover, curcumin exhibited a synergistic antimicrobial effect with antibiotics and antifungals against various pathogens, including methicillin-resistant *S. aureus* [26], *Pseudomonas aeruginosa* [27], enterotoxigenic *Escherichia coli* (ETEC) [28], and *Candida albicans* [29]. Curcumin, with its strong anti-inflammatory properties and anti-*Helicobacter pylori* activity, was also considered in the treatment of *H. pylori*-related gastritis, peptic ulcers, and gastric adenocarcinoma [6].

Despite various studies concerning the antibacterial and antifungal properties of curcumin, insufficient data exist on its effects against different strains of microorganisms, especially clinical isolates and multidrug-resistant (MDR) ones. Additionally, the minimum inhibitory concentrations (MICs) of this natural plant substance against planktonic forms of many common human pathogens have not yet been determined. In turn, this activity of curcumin against such bacteria as *A. lwoffii* [20], *Proteus mirabilis* [24,30], *Serratia marcescens* [20,24], *Stenotrophomonas maltophilia* [31], and *Streptococcus agalactiae* [30] has been examined sporadically. In the contemporary research, the in vitro ability of curcumin to inhibit microbial growth has frequently been tested against a small (4–6) number of species representing a small group of taxa, mostly *E. coli*, *P. aeruginosa*, and *S. aureus*, and less often *Bacillus subtilis* and *Enterococcus faecalis* (e.g., [32,33,34,35,36,37,38]). Some works have reported the minimum inhibitory concentration (MIC) value for only one species and a single, usually reference, strain (e.g., [39,40,41,42]). Sometimes, too low concentrations of curcumin have been used to determine its antimicrobial activity, for instance, at the maximum levels of 64 µg/mL [38], 100 µg/mL [20], 128 µg/mL [33], 156 µg/mL [37], 256 µg/mL [43], 330 µg/mL [28], and 375 µg/mL [44]. Hence, there is still a need for extensive research of the effects of curcumin against a large number of microbial strains and species by a standardized method. The broth microdilution assay is a widely used method that gives the opportunity to compare obtained results with the literature data.

The objective of this study was to assess the antimicrobial efficacy of curcumin against various strains of bacteria and yeast-like fungi. More than 100 strains, mainly clinical isolates, belonging to 19 species were tested in vitro. For the research, common human pathogens including those causing infections of skin, chronic wounds, and mucous membranes were selected. Many of them are also responsible for the opportunistic and hospital-acquired infections (HAIs). The activity of curcumin was determined against 16 MDR strains of *Staphylococcus aureus*, *S. haemolyticus*, *Enterococcus faecalis*, *Escherichia coli*, and *Proteus mirabilis*.

## 2. Results

Our research exhibited a strong variation in the biological activity of curcumin depending on the microbial species and strains (Table 1). The minimum inhibitory concentrations (MICs) of this substance ranged from 31.25 to 5000 µg/mL. In some cases, curcumin was inactive at concentrations tested (7.8–5000 µg/mL). It was observed for 28 out of 111 strains investigated (25.2%). The highest differentiation of the MICs between strains was detected for *Pseudomonas aeruginosa* (from 62.5 to >5000 µg/mL), *Enterococcus faecalis* (62.5–5000 µg/mL), and *Staphylococcus aureus* (from 125 to >5000 µg/mL). Only for methicillin-sensitive *Staphylococcus haemolyticus* (6 strains), *Acinetobacter baumannii* (3 strains), and *Candida glabrata* (2 strains) were no differences in the MIC level found. In turn, *Klebsiella pneumoniae* showed a little variation in this respect. For five clinical isolates of this pathogen, curcumin was effective at a concentration of 2000 µg/mL and for one strain at 3000 µg/mL. In the case of all other species, a significant (usually large) differentiation in the antimicrobial efficacy of curcumin against individual strains was determined (Table 1). Nevertheless, some regularities observed for microbial species and their groups can be pointed out here.

First of all, a clearly stronger effect of curcumin against Gram-positive than Gram-negative bacteria was determined (Table 1). The median value of MICs for the above-mentioned groups of microbes reached 500 and 2000 µg/mL, respectively. In addition, the strain-specific activity of curcumin and its effect on individual species were interesting. For example, this plant molecule very strongly inhibited the growth of one strain of *P. aeruginosa* (MIC = 62.5 µg/mL), while for five clinical isolates and two reference strains of this species it showed poor activity with MICs in the range of 2000 and >5000 µg/mL (Table 1). For most bacterial strains tested (65/97, 67.0%), curcumin exhibited low antimicrobial efficacy (MIC = 1000–5000 µg/mL) or was inactive (>5000 µg/mL). However, 16 bacterial strains (16.5%) had a very high (MIC = 31.25–62.5 µg/mL) or high (125–250 µg/mL) sensitivity to this natural compound.

The median MICs calculated for individual species demonstrated very high or high effects of curcumin against clinical strains of *Streptococcus pyogenes* (median MIC = 31.25 µg/mL), methicillin-sensitive *Staphylococcus aureus* (250 µg/mL), and *Acinetobacter lwoffii* (250 µg/mL). However, other taxa from these genera, namely *Streptococcus agalactiae* (median MIC = 2500 µg/mL), *Staphylococcus epidermidis* (1000 µg/mL), *S. haemolyticus* (500 µg/mL), and *Acinetobacter baumannii* (>5000 µg/mL) showed a significantly lower sensitivity. In addition, low or no biological effects of curcumin were observed in the case of methicillin-resistant strains of *S. aureus* (median MIC >4500 µg/mL) and *S. haemolyticus* (>5000 µg/mL). A similar regularity was found for Gram-negative bacteria. The median MICs of the multidrug-resistant (MDR) strains ESβL-positive *Escherichia coli* and ESβL-positive *Proteus mirabilis* reached 4500 and >4500 µg/mL, respectively, while for other clinical strains of these pathogens the median MICs were 1500 and 3000 µg/mL, respectively (Table 1).

Although the multidrug-resistant types of *S. aureus*, *S. haemolyticus*, *E. coli*, and *P. mirabilis* exhibited some variation in their sensitivity to curcumin, the values of MICs were not less than 2000 µg/mL. In 6 cases out of 16 MDR strains (37.5%), this plant molecule was inactive at concentrations tested (MIC > 5000 µg/mL). In like manner also, antifungal properties of curcumin for 14 tested strains of *Candida* spp. and *Saccharomyces cerevisiae* were shown to be low. The median MIC reached above 5000 µg/mL, and no significant differentiation in this respect was demonstrated for most strains of yeast-like fungi (Table 1).

## 3. Discussion

Previous studies have shown a broad spectrum of antimicrobial properties of curcumin by various pharmacological points of action [45]. It has been found that curcumin can inhibit bacterial DNA replication and alter gene expression. Moreover, it damages the bacterial cell membrane and reduces the motility of microorganisms [46]. The in vitro studies have shown that curcumin inhibits the polymerization of FtsZ protofilaments and disturbs the GTPase activity in cytoskeleton of *B. subtilis*, *E. coli* [47], and *S. aureus* [26]. Through this mechanism, it can influence the cell division and proliferation of bacteria. Other investigations have exhibited that curcumin stimulates an apoptosis-like response in *E. coli* [41]. In turn, Alalwan et al. [48] observed its anti-adhesive effects against *C. albicans* biofilm formation at the sub-inhibitory concentration of MIC/2. The antibiofilm properties of curcumin have also been reported for various bacterial species, including *A. baumannii* [42], *E. faecalis* [39], *E. coli*, *P. aeruginosa*, *P. mirabilis*, *S. marcescens* [24], *S. epidermidis* [49], and *S. mutans* [50].

Our findings showed a significantly higher activity of curcumin against Gram-positive than Gram-negative bacteria (median MIC = 500 versus 2000 µg/mL). This regularity was observed by Lutomski et al. [20] and some other authors [32,37,51], although the small number of bacterial strains and species analyzed in the contemporary research did not allow the drawing of certain conclusions in this respect. It is thought that the stronger effect of curcumin and other natural substances against Gram-positive bacteria results from the differences in the structure and composition of microbial cell walls [52]. The cells of Gram-positive bacteria are surrounded by a thick peptidoglycan layer with an additional class of lipoteichoic acids (LTA), but they do not have an outer membrane (OM). The OM of Gram-negative bacteria is largely responsible for their resistance to a broad spectrum of antibiotics, such as β-lactams, quinolones, and colistins [53].

Curcumin exhibited a strong effect on clinical isolates of *S. pyogenes* (MIC = 31.25, 62.5, 125 µg/mL), *A. lwoffii* (125, 250 µg/mL), and methicillin-sensitive *S. aureus* (125, 250, 500 µg/mL). Other authors have also reported a high sensitivity of these pathogens to curcumin, although the above-mentioned species have been examined to varying degrees. A lot of data exists for *S. aureus* (e.g., [20,32,34,35,37,54]), while in the case of *S. pyogenes* [20,31,33,55], and *A. lwoffii* [20], they are few. The very strong antimicrobial activity of curcumin, probably due to the use of acetone as a solvent, was shown in the studies of Lutomski et al. [20]. The MIC values reached 5, 15–30, and 20–40 µg/mL for *A. lwoffii*, *S. pyogenes*, and *S. aureus*, respectively. Betts et al. [31] observed the inhibitory effect of curcumin on the growth of three wound isolates of *S. pyogenes* at the concentrations of 64–128 µg/mL. In turn, Gómez et al. [55] determined the MICs of four clinical strains of this species as 128 µg/mL, whereas according to Wang et al. [33] it was >128 µg/mL for a penicillin-susceptible strain. Recent investigations have displayed that the MICs of curcumin against reference strains of *S. aureus* range between 125 and 500 µg/mL (e.g., [32,35,37,54]).

In addition to six clinical isolates of methicillin-sensitive *S. aureus* (MSSA), we tested the activity of curcumin against one MSSA reference strain—*S. aureus* ATCC 29213 with the MIC of 250 µg/mL, which was in accordance with the earlier results obtained for this strain at the level of 219 µg/mL [34] and >128 µg/mL [33]. In the study cited above [34], the effect of curcumin was also determined for methicillin-resistance *S. aureus* ATCC 43300, giving a similar score (MIC = 217 µg/mL). Comparable values of the MICs were also shown by Mun et al. [56] for clinical MRSA isolates (MIC = 125, 250 µg/mL). Moreover, no differences were found in this respect (MIC = 250 µg/mL) for two reference strains: *S. aureus* ATCC 33591 (MRSA) and *S. aureus* ATCC 25923 (MSSA). In this context, it is interesting that our investigations displayed a poor activity of curcumin against clinical strains of MRSA (MIC = 2000, 4000, >5000 µg/mL), methicillin-resistant *S. haemolyticus* (MIC = 2000, >5000 µg/mL), and vancomycin-resistant *E. faecalis* (MIC = 5000 µg/mL), much less than for non-multidrug-resistant (NMDR) isolates (Table 1). The MIC value for NMDR *S. haemolyticus* was 500 µg/mL, while for *E. faecalis* it ranged from 62.5 to 2000 µg/mL (median MIC = 500 µg/mL). To our knowledge, the activity of curcumin against *S. haemolyticus* has not yet been studied, whereas the previous results for *E. faecalis* were similar to our findings. The MIC of reference strains of this species varied between 156 [37] and 625 µg/mL [39,40] for *E. faecalis* ATCC 51299 and *E. faecalis* ATCC 29212, respectively.

Among the Gram-negative bacteria, most of the strains tested in our work showed a weak sensitivity to curcumin (Table 1). The exceptions were individual strains of *P. aeruginosa* (MIC = 62.5 µg/mL) and *Klebsiella oxytoca* (MIC = 500 µg/mL), two clinical isolates of *E. coli* (MIC = 500 µg/mL), and also three strains of *A. lwoffii* (MIC = 125–250 µg/mL). However, for other species (*A. baumannii*) from the last genus, the MIC reached >5000 µg/mL. Betts and Wareham [43] investigated the activity of curcumin on eight MDR clinical isolates and the antibiotic susceptible *A. baumannii* ATCC 19606, but a too low concentration of the plant substance did not allow for a precise determination of its antibacterial effect with the MIC > 256 µg/mL. For the same reason, the MICs of curcumin against *A. baumannii* ATCC BAA-1605 and *A. baumannii* ATCC 17978 were exhibited to be >64 µg/mL [38] and >500 µg/mL [42], respectively. In the next work of the authors cited above [31], the MICs of curcumin were reported as 512–1024 µg/mL.

In light of our research, the case of *P. aeruginosa* is of particular interest. Only one clinical isolate of this pathogen displayed a very strong sensitivity to curcumin (MIC = 62.5 µg/mL), while the MICs were very high, ranging from 2000 to above 5000 µg/mL for other strains tested. Previous investigations have shown that curcumin may exhibit a strong or moderate effect on reference strains of *P. aeruginosa* with the MICs from 30 to 500 µg/mL [32,35,57,58]. The most literature data exists for two reference strains of *P. aeruginosa*, namely PAO1 and ATCC 27853, but they provide varied and sometimes imprecise results. The MICs of curcumin against *P. aeruginosa* PAO1 were determined as 30–512 µg/mL [27,31,57,59]. For *P. aeruginosa* ATCC 27853 it was 73.7–512 µg/mL [31,34,58], but some authors reported the MICs values as >64 µg/mL [38], >128 µg/mL [33], or >375 µg/mL [44]. In turn, our studies did not show significant activity of curcumin on this strain with a MIC of 5000 µg/mL. A comparison of findings from present and earlier works indicates that *P. aeruginosa* is characterized by a large differentiation in sensitivity to curcumin, and strains resistant to this natural substance may arise. It is consistent with common observations of a high resistance of this pathogen to antibiotics, including fluoroquinolones, β-lactams, and carbapenems [59].

Interesting results have also been provided by studies concerning *E. coli*. We determined a moderate effect of curcumin (MIC = 500 µg/mL) against only two clinical isolates, while other strains exhibited a poor sensitivity to this compound (MIC = 1000–3000 µg/mL). Curcumin showed weaker activity against clinical MDR isolates of ESβL-positive *E. coli* (MIC = 2000–5000 µg/mL). Numerous research on reference strains have shown the very strong, strong, and medium effects of curcumin on this bacterium with MICs of 12–74.3 µg/mL [41,60], 120–192 µg/mL [24,61], and 300–500 µg/mL [32,62]. Most often, the results have been reported for a commensal *E. coli* strain ATCC 25922. They ranged from 163 [34] to 302 µg/mL [58], but some authors determined the MICs as >64 µg/mL [38], >128 µg/mL [33], >375 µg/mL [44], and >500 µg/mL [51]. Our investigations for this reference strain exhibited even a lower activity of curcumin at the MIC level of 2000 µg/mL.

Similarly to most strains of Gram-negative bacteria tested in the preset work, yeast-like fungi also displayed poor sensitivity to curcumin (Table 1). The MICs for clinical isolates of *C. albicans* varied between 1000 and >5000 µg/mL. In the case of *C. glabrata* and *C. tropicalis*, curcumin was inactive at concentrations tested (MIC > 5000 µg/mL), while for *S. cerevisiae* the MIC was 5000 µg/mL. Recent studies of Narayanan et al. [63] have shown the activity of curcumin against *C. albicans* ATCC 90028 and *C. glabrata* ATCC 90030 at the level of 500 µg/mL, whereas for the clinical isolate of *C. albicans* it was 2000 µg/mL. The differences between ATCC and clinical strains of yeast-like fungi were also reported in the earlier work of Neelofar et al. [64]. The MICs of reference strains reached 250–500 and 500 µg/mL for *C. albicans* and *C. tropicalis*, respectively. In turn, for clinical isolates it was 250–2000 and 500–1000 µg/mL, respectively. No differentiation in this respect was found in two strains of *C. glabrata*: ATCC 90030 and a cutaneous clinical isolate (MIC = 500 µg/mL). According to the authors cited above [63], the greater sensitivity of standard *Candida* strains is caused by the suppression of virulence factors during a repeated subculture of the laboratory strains. This regularity has been also exhibited for some bacteria [65].

## 4. Materials and Methods

### 4.1. Curcumin

Curcumin from *Curcuma longa* L. (cat. no. C1386, Figure 1) used in the present work was purchased from Sigma-Aldrich, Poland. This natural plant substance was dissolved in a 15% water solution of dimethyl sulfoxide (DMSO) obtained from Sigma-Aldrich (Poznań, Poland), in a final concentration of 10 mg/mL. In addition, 15% DMSO was used as a negative control.

### 4.2. Microbial Strains and Culture Media

Antimicrobial activity of curcumin was investigated against growth in aerobic conditions of six species of Gram-positive bacteria (*Enterococcus faecalis*, *Staphylococcus aureus*, *S. epidermidis*, *S. haemolyticus*, *Streptococcus agalactiae*, *S. pyogenes*), nine Gram-negative bacteria (*Acinetobacter baumannii*, *A. lwoffii*, *Escherichia coli*, *Klebsiella oxytoca*, *K. pneumoniae*, *Proteus mirabilis*, *Pseudomonas aeruginosa*, *Serratia marcescens*, *Stenotrophomonas maltophilia*), and four species of yeast-like fungi (*Candida albicans*, *C. glabrata*, *C. tropicalis*, *Saccharomyces cerevisiae*). Mostly, six clinical strains were examined for each microbial species (Table 1). Additionally, four reference strains, namely *S. aureus* ATCC 29213, *E. coli* ATCC 25922, *P. aeruginosa* ATCC 27853 (Boston 41501), and *P. aeruginosa* NCTC 6749 were used as controls. From among the multidrug-resistant (MDR) bacteria, we in vitro tested 16 clinical isolates of methicillin-resistant *S. aureus* (MRSA), methicillin-resistant *S. haemolyticus* (MRCNS), vancomycin-resistant *E. faecalis* (VRE), extended-spectrum β-lactamases (ESβL) positive *E. coli*, and ESβL-positive *P. mirabilis*. The total number of strains investigated was 111 from 19 species. The microbial strains were obtained from the collection of the Chair and Department of Medical Microbiology at Poznań University of Medical Sciences (Poland). The clinical strains were isolated from the skin and mucous membrane infections. Their drug resistance was tested according to the European Committee on Antimicrobial Susceptibility Testing (EUCAST) [66].

The microorganisms were grown at 35 °C for 24 h, bacteria in tryptone soy agar (TSA; Graso Biotech, Starogard Gdański, Poland), and fungi in Sabouraud dextrose agar (SDA; Graso Biotech, Starogard Gdański, Poland).

### 4.3. Antimicrobial Activity

The minimum inhibitory concentrations (MICs) of curcumin were determined by the broth microdilution method using 96-well plates (Nest Scientific Biotechnology, Wuxi, China). The in vitro tests were carried out according to the recommendations of the Clinical and Laboratory Standards Institute (CLSI) [67] and EUCAST method [66], and were described in our previous publication [52]. Primarily, 90 µL of Mueller–Hinton broth (Graso Biotech, Starogard Gdański, Poland) was placed in each well. The following final concentrations of curcumin were obtained: 5000, 4000, 3000, 2000, 1000, 500, 250, 125, 62.5, 31.25, 15.6, and 7.8 µg/mL.

The inoculums were adjusted to contain approximately 10^8^ CFU/mL of microorganisms. Then, 10 µL of the proper inoculums were added to the wells, obtaining a final concentration of 10^5^ CFU/mL. To each well, 10 μL of a 1% aqueous solution of 3-(4,5-dimethyl-2-thiazolyl)-2,5-diphenyl-2*H*-tetrazolium bromide (MTT), purchased from Sigma-Aldrich (Poznań, Poland), was added [65]. Next, the plates were incubated at 35 °C for 24 h. The MIC of curcumin was taken as the lowest concentration of this substance that inhibited any visible microbial growth. The analyses were repeated two times for all strains tested.

### 4.4. Statistical Analysis

The mean MIC values of curcumin against all microbial strains tested are shown in Table 1. For individual species and their groups, medians were calculated. The Kruskal–Wallis and post-hoc tests were applied to determine the statistical significance of differences in the MICs of Gram-positive and Gram-negative bacteria, fungi, and multidrug-resistant bacteria. The results were considered significant at the level of *p* < 0.05. Data were tested using Statistica for Windows software.

## 5. Conclusions

Our study exhibited a broad spectrum of antimicrobial activity of curcumin. The in vitro tests included over 100 bacterial and fungal strains belonging to 19 species. To our knowledge, the minimum inhibitory concentrations (MICs) of this natural plant compound against planktonic forms of *Klebsiella oxytoca* and *Staphylococcus haemolyticus* were determined for the first time. Similarly, the effects of curcumin on methicillin-resistant *S. haemolyticus* (MRCNS), vancomycin-resistant *Enterococcus faecalis* (VRE), extended-spectrum β-lactamases (ESβL) positive *Escherichia coli*, and ESβL-positive *Proteus mirabilis* had not yet been studied.

The obtained results confirmed the much greater sensitivity to curcumin of Gram-positive than Gram-negative bacteria. The present study displayed a significantly larger variation in the MIC values of this chemical compound than the previous works and suggests that numerous clinical strains of widespread pathogens are poorly sensitive to curcumin. Similarly, the high MICs of multidrug-resistant isolates of *Staphylococcus aureus* (MRSA), *S. haemolyticus*, *Escherichia coli*, and *Proteus mirabilis* were determined. Our findings also imply poor activity of curcumin against clinical isolates of *Candida* spp. Nevertheless, curcumin exhibited strong antibacterial properties in the in vitro tests, and it was very effective against some species and strains: *Streptococcus pyogenes*, methicillin-sensitive *S. aureus*, *Acinetobacter lwoffii*, and individual strains of *Enterococcus faecalis* and *Pseudomonas aeruginosa*.

The efficacy of curcumin varied widely depending on the microbial species and strain. Moreover, the sensitivity of particular bacterial species was not associated with its affiliation to the genus, and it could differ a lot, as in the case of *S. pyogenes* and *S. agalactiae* as well as *A. lwoffii* and *A. baumannii*. Hence, curcumin can be considered as a promising antibacterial agent, but with very selective activity towards individual species and strains.

The long tradition of use of *Curcuma longa* in folk medicine and cuisine, and the numerous in vitro, in vivo, and clinical trials concerning the activity of curcumin have confirmed its pleiotropic pro-health and therapeutic properties. The present in vitro tests exhibited the strong or moderate effect of this phytochemical on some Gram-positive and Gram-negative pathogens. The literature data and our findings allow us to state that curcumin may have a therapeutic potential in the treatment of skin and chronic wound infections (*S. pyogenes*, *S. aureus*, *A. lwoffii*), urinary tract infections (*E. coli*, *P. aeruginosa*, *P. mirabilis*, *Serratia marcescens*), and root canal infections (*E. faecalis*). The present results show the health benefits of using of curcumin as an important spice and food additive with not only its coloring, flavoring, and preservative properties, but also with the antimicrobial activity against human pathogens. A stronger activity of curcumin against Gram-positive than Gram-negative bacteria observed in our investigations and by some other authors is very interesting from a practical point of view and requires further detailed studies because numerous Gram-positive pathogens, including *S. pyogenes*, *S. aureus*, *S. haemolyticus*, *S. epidermidis*, and *E. faecalis*, are still an ongoing challenge for healthcare in the treatment of infections. Our research does not end the scientific cycle on evaluation of curcumin activity, but it opens the scientific window to the genetic investigations.

## Figures and Tables

**Table 1:** Antimicrobial activity of curcumin against clinical and reference strains of pathogens.

Microbial Strains	Minimum Inhibitory Concentration, Mean MIC Values (µg/mL)	Median
**1. Gram-positive bacteria**		
*Streptococcus pyogenes* (*n* = 6) ^1^	**31.25** (4×) ^2^, **62.5**, 125	31.25
*S. agalactiae* (*n* = 6)	2000 (3×), 3000 (2×), 4000	2500
*Staphylococcus aureus* (*n* = 6)	125, 250 (3×), 500 (2×)	250
*S. aureus* ATCC 29213	250	
*S. haemolyticus* (*n* = 6)	500 (6×)	500
*S. epidermidis* (*n* = 6)	500 (2×), 1000 (3×), 2000	1000
*Enterococcus faecalis* (*n* = 6)	**62.5**, 500 (3×), 1000, 2000	500
Methicillin-resistant *S. aureus* (*n* = 4)	2000, 4000, >5000 (2×)	>4500
Methicillin-resistant *S. haemolyticus* (*n* = 3)	2000, >5000 (2×)	>5000
Vancomycin-resistant *E. faecalis* (*n* = 1)	5000	
Median	500 ^3^ a, >5000 ^4^ b	
**2. Gram-negative bacteria**		
*Acinetobacter lwoffii* (*n* = 3)	125, 250 (2×)	250
*A. baumannii* (*n* = 3)	>5000 (3×)	>5000
*Escherichia coli* (*n* = 6)	500 (2×), 1000, 2000 (2×), 3000	1500
*E. coli* ATCC 25922	2000	
*Klebsiella oxytoca* (*n* = 6)	500, 1000, 2000 (2×), >5000 (2×)	2000
*K. pneumoniae* (*n* = 6)	2000 (5×), 3000	2000
*Pseudomonas aeruginosa* (*n* = 6)	**62.5**, 2000 (2×), 3000, 5000, >5000	2500
*P. aeruginosa* ATCC 27853 (Boston 41501)	5000	
*P. aeruginosa* NCTC 6749	>5000	
*Proteus mirabilis* (*n* = 6)	1000, 2000 (2×), 4000, >5000 (2×)	3000
*Serratia marcescens* (*n* = 4)	2000 (2×), >5000 (2×)	>3500
*Stenotrophomonas maltophilia* (*n* = 1)	>5000	
ESβL-positive *E. coli* (*n* = 4)	2000, 4000, 5000 (2×)	4500
ESβL-positive *P. mirabilis* (*n* = 4)	2000, 4000, >5000 (2×)	>4500
Median	2000 b, >4500 b	
**3. Yeast-like fungi**		
*Candida albicans* (*n* = 10)	1000, 2000, 5000, >5000 (7×)	>5000
*C. glabrata* (*n* = 2)	>5000 (2×)	>5000
*C. tropicalis* (*n* = 1)	>5000	
*Saccharomyces cerevisiae* (*n* = 1)	5000	
Median	>5000 b	
**Negative control**		
15% DMSO	>5000	

^1^ Total number of microbial strains tested, ^2^ the number of strains with an equal sensitivity to curcumin, ^3^ the median for all strains from the group, ^4^ the median for multidrug-resistant strains. The medians with the same letter are not significantly different (post-hoc test, *p* > 0.05). All MIC tests were repeated two times. The lowest MICs are marked with a bold font.
